# CircRNA circ_0006677 Inhibits the Progression and Glycolysis in Non–Small-Cell Lung Cancer by Sponging miR-578 and Regulating SOCS2 Expression

**DOI:** 10.3389/fphar.2021.657053

**Published:** 2021-05-13

**Authors:** Bo Yang, Fang Zhao, Lei Yao, Zhenfeng Zong, Li Xiao

**Affiliations:** ^1^ Department of Thoracic Surgery, Cangzhou Central Hospital, Hebei, China; ^2^ Department of Hematology, Cangzhou Central Hospital, Hebei, China; ^3^ Department of Oncology, Zhongshan Hospital Xiamen University, Xiamen, China

**Keywords:** non–small-cell lung cancer, circ_0006677, microRNA-578, SOSC2, lung cancer

## Abstract

**Objective:** Circular RNAs (circRNAs) have been demonstrated in playing an important role in the physiological and pathological processes (such as cancer). This paper aims to clarify the role of Circ_0006677 in non–small-cell lung cancer (NSCLC) progression.

**Methods:** Using clinical data and *in vitro* cell line models, we revealed the tumor-suppressive role of circ_0006677 in lung cancer. Using the online bioinformatics tool, we predicted the target of circ_0006677 and further validated its regulatory mechanisms responsible for its tumor suppressor function in NSCLC.

**Results:** Circ_0006677 expression was reduced in NSCLC tissues of patients and lung cancer cells in comparison to adjacent normal tissues. Lower expression of circ_0006677 was significantly associated with poorer patient survival. Overexpression of circ_0006677 significantly inhibited the ability of NSCLC cell proliferation, migration, invasion, and glycolysis. Mechanically, circ_0006677 could inhibit NSCLC progression and glycolysis by regulating the expression of the signal transducer inhibitor SOSC2 through sponging microRNA-578 (miR-578).

**Conclusion:** Circ_0006677 prevents the progression of NSCLC *via* modulating the miR-578/SOSC2 axis.

## Highlights

1. Circ_0006677 acts as a tumor suppressor in NSCLC progression
2. Circ_0006677 works as a sponge for miR-578 in NSCLC cells
3. Circ_0006677 inhibits NSCLS *via* the miR-578/SOSC2 axis

## Introduction

Lung cancer is the leading cause of cancer-related deaths worldwide (Sève et al., 2010). Non-small-cell lung cancer (NSCLC) is the most frequently diagnosed pathological type of lung cancer (Sève et al., 2010). Despite recent advances in chemotherapy, targeted therapy, and immunotherapy of NSCLC clinical treatment, the prognosis for NSCLC patients remains poor, and the overall 5-year survival rate is around 17% (Bray et al., 2018). Therefore, a deeper understanding of the mechanisms that modulate the tumorigenesis and progression of NSCLC would be important to improve the diagnosis and treatment of this cancer.

Circular RNAs (circRNAs) represent a large class of endogenous RNAs with covalently closed continuous loop (Chen and Yang, 2015). With the development of RNA sequencing technologies and bioinformatics, the abundance and diversity of circRNAs have been identified, and their expression patterns have been identified in various developmental stages and physiological conditions. Recently, circRNAs were shown to regulate multiple biological processes by functioning as microRNA (miRNA) sponges (Hansen et al., 2013; Wan et al., 2016), interacting with RNA-binding proteins (Du et al., 2016), mediating gene transcription (Li et al., 2015) and protein translation (Abe et al., 2015; Mentha et al., 2019). Abnormal expression of circRNAs has been reported in many cancers and is involved in the regulation of cancer progression (Meng et al., 2017; Bach et al., 2019). For instance, circ-ITCH is upregulated in NSCLC tissues and suppresses NSCLC cell proliferation *via* the Wnt/β-catenin signaling pathway by acting as a sponge for miR-7 and miR-214 (Wan et al., 2016). However, the functions of a large number of circRNAs in NSCLC remain to be clarified. In addition, dysregulation of miRNAs is also associated with NSCLC progression (Uchino et al., 2013). In many types of cancer, miR-578 was reported to promote cancer development (Chen et al., 2020; Ji et al., 2020; Wu et al., 2020). SOCS2 is a member of the suppressor of cytokine signaling (SOCS) family and represses the cytokine-induced signaling transduction, thus inhibiting cancer progression (Ricobautista et al., 2006; Das et al., 2017; Chhabra et al., 2018; Tong et al., 2019).

Here, we found that circ_0006677 is significantly downregulated in NSCLC tissues. We further showed that circ_0006677 inhibits NSCLC cell proliferation, migration, invasion, and glycolysis *in vitro* and represses tumor growth in the xenograft mouse models. Mechanically, circ_0006677 functions as a sponge of miR-578 to induce the expression of SOCS2, thereby suppressing NSCLC progression and glycolysis. Therefore, circ_0006677 may be a promising biomarker for NSCLC diagnosis and a potential therapeutic candidate for NSCLC treatment.

## Materials and Methods

### Patient Samples

A total of 88 NSCLC patients whose diagnoses were confirmed *via* biopsy were selected for this study. These patients received no prior radiotherapy or chemotherapy before surgery. This study was approved by the Research Ethics Committee at Cangzhou Central Hospital. All patients signed the informed consent for the use of their patient information and tissues. Tumor tissues and adjacent noncancerous tissues were collected and stored at 80°C until use.

### Database Analysis of Circ_0006677 Expression in Non-Small-Cell Lung Cancer

We compared the levels of circ_0006677 in NSCLC tissues and adjacent normal tissues using the GEO dataset (GSE112214).

### Bioinformatic Analysis

The potential miRNAs that might interact with circ_0006677 was predicted using two online tools (circBank and CircInteractome). The interaction between miRNA and its target genes was analyzed using the TargetScan database.

### Cell Culture

NSCLC cell lines (CALU3, CALU6, A549, H1229, and H1975) and human bronchial epithelial cells (HBE) were purchased from the Cell Bank of Type Culture Collection of Chinese Academy of Sciences, Shanghai, China). All these cell lines were cultured in RPMI-1640 medium (Solarbio, China) supplemented with 10% fetal bovine serum, streptomycin, and penicillin at 37°C in 5% CO2. HEK293T cells were cultured in DMEM supplemented with 10% fetal bovine serum, 100 U/ml penicillin (SH30010, Hyclone), and 100 mg/ml streptomycin.

### Lentiviral Vector Construction

To generate circ_0006677 overexpression lentiviral plasmid, circ_0006677 was constructed into a pLV plasmid. For virus packaging, pLV-circ_0006677, PsPAX2, and pMD2G plasmids were co-transfected into HEK293T cells. The viral supernatant was collected to construct stable circ_0006677–overexpressing cell lines.

### RNase R Treatment

RNase R (Epicentre, China) was used to eliminate the linear RNAs. The expression of GAPDH and circ_0006677 before and after RNase R treatment was assessed.

### Cell Proliferation Assay

Cells growth was monitored with CCK-8 reagent every day following the manufacturer’s instruction (Dojindo Laboratories, Kumamoto, Japan). In brief, 1, 000 cells were seeded in 96-well plates and cultured overnight. CCK-8 reagent was added at the time point, and the absorbance at 450 nm was measured using a DNM-9602 Microplate Reader (Perlong, Beijing, China).

### Colony Formation Assay

One thousand cells were seeded in six-well plates and cultured for 14 days. Cell colonies were fixed and stained with 0.5% crystal violet in methyl alcohol. Images of cell clones were taken, and colonies were counted with ImageJ.

### Migration and Invasion Assay

For migration assay, cells in the logarithmic growth stage were starved for 24 h, and the cells were digested the next day, centrifuged, and resuspended for a final concentration of 1 × 10 (Wan et al., 2016)/ml. Cell suspension (0.2 ml) was added to the Transwell upper chamber, and 700 μl of precooled DMEM cell culture medium containing 10% FBS was added to the lower chamber. The cells were cultured in a cell incubator containing 5% CO2 at 37°C. After 24 h, the Transwell chamber was removed and the cells in the upper chamber and the basement membrane were swabbed with wet cotton swabs. The cells were fixed with methanol for 30 min and stained with 0.1% crystal violet dye for 20 min. Five fields (100×) were randomly selected to count the number of transmembrane cells. For invasion assay, Matrigel was loaded in Transwell upper chambers and curdled at 37°C before cells were added to the upper chambers. After culture for 24 h, cells on the lower surface of the membrane were fixed with 4% paraformaldehyde for 10 min and stained by 0.1% crystal violet for 30 min. Cells were counted using an inverted microscope (magnification, ×20).

### Assay of Glucose Consumption and Lactic Acid Production

Glucose consumption and lactic acid production of the cells were measured by glucose assay and lactate assay following the manufacturers’ instruction (Abcam, United States). In brief, cells were incubated with 2-deoxy-d-glucose (2-DG) for 20 min at 37°C. Cells were then washed with PBS to remove exogenous 2-DG. Cells were lysed with extraction buffer, heated at 85°C for 40 min, cooled on ice for 5 min, and centrifuged at 13,000g. The supernatant was transferred to new tubes and incubated with mix A for 1 h at 37°C. After mix B was added to the reaction mixture, the absorbance of each well was analyzed under the wavelength of 450 nm every 2–3 min on a microplate reader, set in the kinetic mode.

### qRT-PCR Analysis

Samples of patient tissue and NSCLC cell lines were lysed with TRIzol reagent (Invitrogen Carlsbad, CA, United States) and total RNAs were isolated following the manufacturer’s instructions. The RNA sample was reverse transcribed by the Reverse Transcriptase Kit (Takara, Beijing, China). Real-time PCR was performed with the CFX Connect Real-time PCR Machine (Bio-Rad) and SYBR green reagent, as described previously (Zou et al., 2020). GAPDH was used as internal controls. Primer sequences were as follows: MIR578-Forward: CTTCTTGTGCTCTAGGAT and MIR578-Reverse: GAA​CAT​GTC​TGC​GTA​TCT​C; SOCS2-Forward: GGT​CGG​CGG​AGG​AGC​CAT​CC and SOCS2-Reverse: GAA​AGT​TCC​TTC​TGG​TGC​CTC​TT; circ_0006677-Forward: ACT​TGT​GAT​GCC​CTG​ACT​G and circ_0006677-Reverse: ACT​TGG​ATC​CGT​CAC​GTT​G; and GAPDH-Forward: GAA​GGT​GAA​GGT​CGG​AGT​C and GAPDH-Reverse: GAA​GAT​GGT​GAT​GGG​ATT​TC.

### Luciferase Reporter Assay

The wild-type (WT) or mutated (MUT) sequences at 3′-UTR of *SOCS2* mRNA were synthesized and cloned into the pMIR-Reporter Vector (Promega Corporation, Madison, WI, United States). Using Lipofectamine 2000 reagent, A549 and H1299 cells were co-transfected with SOCS2 wild-type 3′UTR or mutant-type *SOCS2* 3′-UTR with miR-578 mimic or control mimic (miR-NC). 48 h after transfection, cells were harvested, and the luciferase activity was measured using a dual luciferase assay (Promega Corporation, Madison, WI, United States). Each experiment was repeated three times.

### RNA Pull-Down Assay

Biotin-labeled RNAs were transcribed using the Biotin RNA Labeling Mix (Promega Corporation, Madison, WI, United States) and T7 RNA polymerase (Promega Corporation, Madison, WI, United States), treated with RNase-free DNase I (Promega, United States), and then purified with the RNeasy Mini Kit (Promega, United States). Transcriptional production of a biotinylated circ_0006677 probe or control probe (NC-probe) was heated at 95°C for 5 min, placed on ice for 5 min, and placed at room temperature for 20 min to form the secondary structure. Folded RNA was mixed with cell extracts for 2 h. Then, 50 µl of streptavidin agarose beads (Invitrogen, United States) were added to each binding reaction and incubated for 1.5 h. After washing the beads, the samples and inputs were digested by Proteinase K, and RNA was isolated and quantified by qRT-PCR assay.

### Western Blot Analysis

Cells were lysed by RIPA lysis buffer (Beyotime, Shanghai, China). Protein concentration was determined using BCA assay (Beyotime). Proteins were subjected to 10% SDS–polyacrylamide gel electrophoresis. Separated proteins were transferred onto polyvinylidene difluoride (PVDF) membranes (Millipore, Billerica, MA, United States) and immunoblotted with primary antibodies: anti-SOCS2 (ab71676, Abcam), anti-GAPDH (ab76523, Abcam), anti-MMP2 (ab97779, Abcam), and anti-MMP9 (ab38898, Abcam), and secondary antibody: goat anti-rabbit (ab205719; Abcam). Bands were visualized using the EasyBlot ECL Kit (Sangon Biotech, China). The intensity of the blots was quantified by ImageJ (Rawak Software, Germany).

### Xenograft Mouse Models

Fifty four-week-old BALB/C^nu/nu^ nude mice (20 g) were purchased from Beijing Vital River Laboratory Animal Technology (Beijing, China) and employed to establish the mouse models, as reported previously (Zhu et al., 2019). In brief, 2 × 10^6^ A549 cells were subcutaneously injected into the nude mice. Tumor’s volume of each mouse was measured every 7 days until 35 days. All procedures and animal experiments were approved by the Animal Ethics Committee of Cangzhou Central Hospital.

### Statistical Analysis

Statistical analyses were performed using SPSS 21.0 software (IBM, United States). All experiments were repeated three times. All the data were presented as mean ± standard deviation (SD). Differences between two groups compared with unpaired two-tailed *t*-test, while ANOVA was used for multiple comparisons. The Kaplan–Meier method was used for survival analysis. All correlations used in this analysis were Pearson’s correlation coefficient. *p* < 0.05 was considered as statistically significant.

## Results

### Circ_0006677 Is Downregulated in NSCLC Tissues and Cell Lines

**FIGURE 1:**
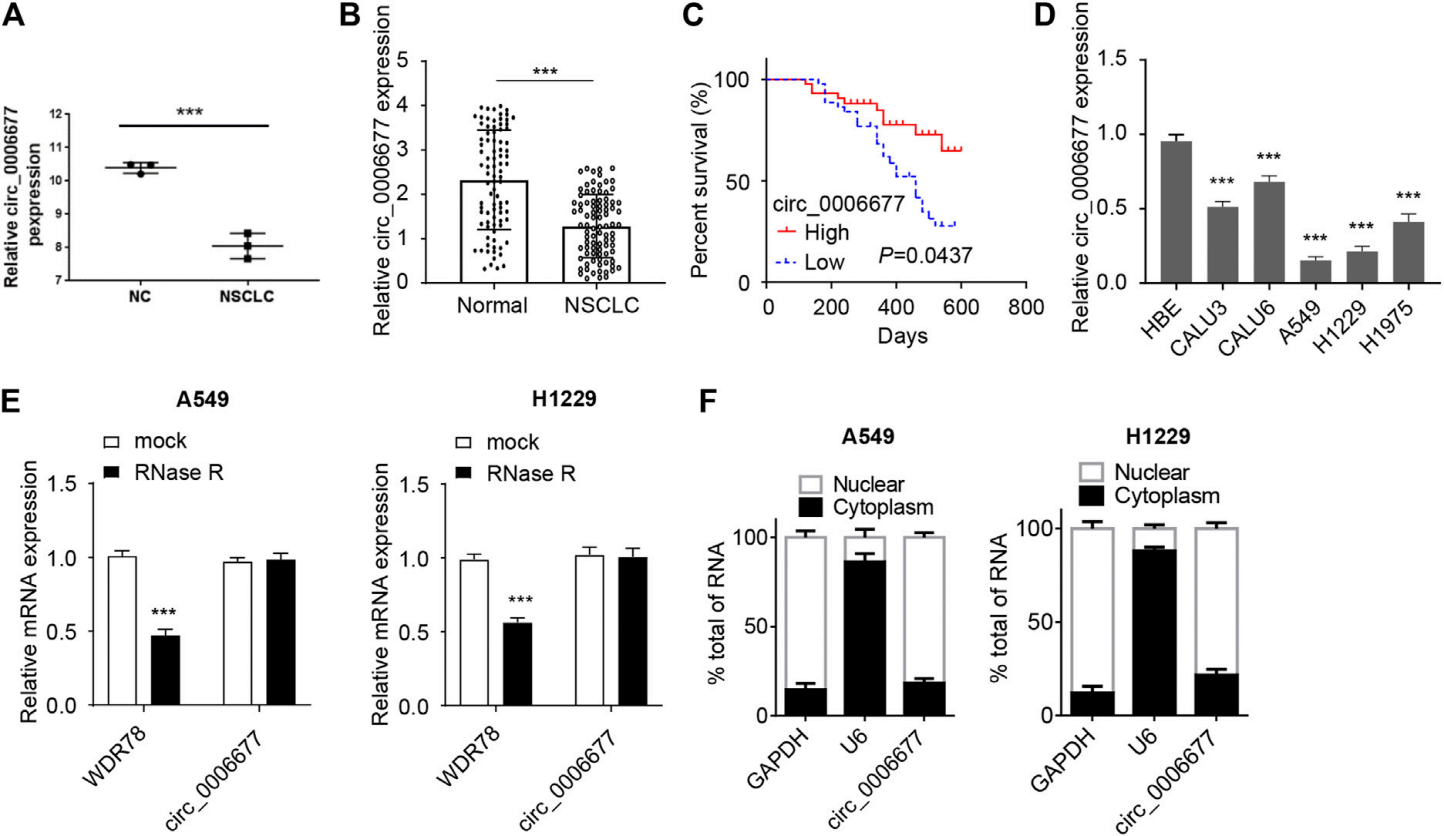
Circ_0006677 is reduced in NSCLC tissues and cells. **(A)** Analysis of the differentially expressed circRNAs in NSCLC tissues and adjacent normal tissues (GSE112214). Three pairs of tumor and respective control tissues were analyzed. **(B)** The expression of circ_0006677 in 88 pairs of NSCLC tissues and adjacent normal tissues was detected using qRT-PCR assays. **(C)** The Kaplan–Meier survival analysis of the correlation between circ_0006677 levels with the prognosis of NSCLC patients. Eighty-eight patients were divided into circ_0006677-high and circ_0006677-low according to the median expression value of circ_0006677 in NSCLC tissues. **(D)** The expression of circ_0006677 in NSCLC cell lines and human bronchial epithelial cells (HBE) were detected with qRT-PCR analysis. **(E)** The expression of WDR78 and circ_0006677 in A549 and H1229 cells after RNase R treatment were detected by qRT-PCR analysis. **(F)** Analysis of the subcellular location of circ_0006677 in A549 and H1229 cells. The cytoplasm and nucleus were separated followed by detecting the circ_0006677 level using qRT-PCR assays. U6 and GAPDH were used as internal references of the nucleus and cytoplasm, respectively. ***p < 0.0001.

To investigate the expression of circ_0006677 in NSCLC tissues, we compared the levels of circ_0006677 in three pairs of NSCLC tissues and adjacent normal tissues in the GEO dataset (GSE112214). We found that circ_0006677 was downregulated in NSCLC tissues compared with adjacent normal tissues (Figure 1A). By performing qRT-PCR analysis, we further confirmed the lower expression of circ_0006677 in NSCLC tissues (*n* = 88) than the adjacent normal tissues (*n* = 88) (Figure 1B). Moreover, according to the median of the expression value, 88 patients were divided into the circ_0006677-high group and the circ_0006677-low group. We found that higher levels of circ_000667 expression were significantly associated with longer overall survival (Figure 1C). Moreover, the expression of circ_0006677 was negatively related to the tumor size, TNM stage, and lymph node metastasis (Table 1). The qRT-PCR assays revealed that the expression of circ_0006677 was significantly lower in a series of NSCLC cell lines (especially, A549 and H1299 cells) than the HBE (Figure 1D). Furthermore, A549 and H1299 cells were treated with RNase R, and the expression of WDR78 and circ_0006677 was detected using qRT-PCR assays. The levels of WDR78 were significantly decreased after RNase R treatment, while the expression of circ_0006677 was not altered (Figure 1E). Nuclear/cytoplasmic fractionation of NSCLC cells was performed to explore the subcellular location of circ_0006677 in NSCLC cells. Our qRT-PCR assays suggested that circ_0006677 was mainly located and enriched in the cytoplasm (Figure 1F). These results indicated that circ_0006677 might play a tumor-suppressive role in NSCLC.

**TABLE 1:** Correlations of circ_0006677 expression with clinicopathologic features of non-small cell lung cancer.

Factor	Circ_0006677 expression		*p* value
	Low (*n* = 44)	High (*n* = 44)	
Age, years			0.286
≤60	24	19	
>60	20	25	
Sex			0.514
Male	28	25	
Female	16	19	
Tumor differentiation		0.197	
I	16	22	
II	28	22	
Tumor size			0.001
≤2 cm	10	26	
>2 cm	32	18	
T classification		0.029	
T1-T2	15	25	
T3-T4	27	17	
N classification		0.016	
N0-N1	17	28	
N2-N3	25	14	
Clinical stage		0.016	
I/II	16	27	
III/IV	26	15	
Lymph node metastasis		0.012	
Yes	22	32	
No	22	10	

### Circ_0006677 Inhibits the Proliferation, Migration, Invasion, and Glycolysis of Non–Small-Cell Lung Cancer Cells

**FIGURE 2:**
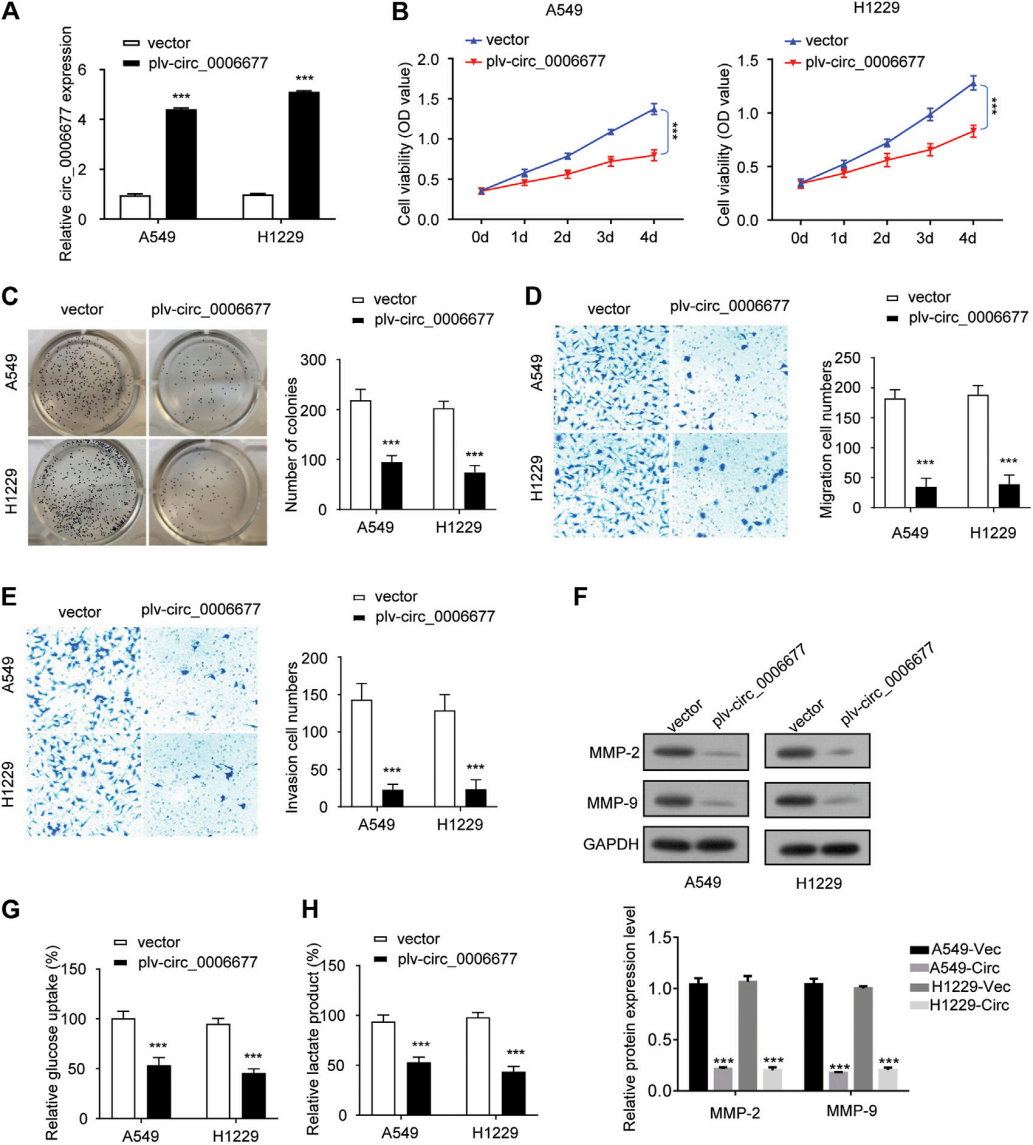
Overexpression of circ_0006677 inhibits the proliferation, migration, invasion, and glycolysis of NSCLC cells. **(A)** A549 and H1299 cells were infected with lentivirus coding circ_0006677 and overexpression of circ_0006677 in A549, and H1299 cells were validated using qRT-PCR analysis. **(B)** Impact of circ_0006677 expression on the proliferation of A549 and H1299 cells was investigated using CCK-8 assays at indicated time points. **(C)** Impact of circ_0006677 expression on colony formation capacity of A549 and H1299 cells. **(D)**. Impact of circ_0006677 expression on cell migration. **(E)** Impact of circ_0006677 expression on cell invasion ability. **(F)** Western blot assay of cell migration/invasion–related proteins in A549 and H1229 cells transfected with a control vector or circ_0006677 vector. Circ: circ_0006677. Lower: quantitative analysis of Western blot data. **(G)** Glucose assay was performed to evaluate the impact of circ_0006677 expression on glucose consumption. **(H)** The effects of circ_0006677 on lactic acid production in A549 and H1299 cells were evaluated using the lactate assay. ***p < 0.0001.

To validate the function of circ_0006677 in NSCLC, we sought to ectopically express circ_0006677 in A549 and H1299 cells, which have the lowest levels of circ_0006677. To dos so, we infected A549 and H1299 cells with lentivirus to construct stable cell lines overexpressing circ_0006677 (designated A549-circ_000667 and H1299-circ_000667). Compared with control cells, the circ_0006677 expression level was significantly increased in A549-circ_000667 and H1299-circ_000667 cells (Figure 2A).

Overexpression of circ_0006677 significantly inhibited the proliferation (Figure 2B) and colony-forming capacity (Figure 2C) of A549 and H1299 cells. We also evaluated the impact of circ_0006677 overexpression on cell migration and invasion ability. Upregulation of circ_0006677 could significantly inhibit the migration and invasion ability of A549 and H1299 cells (Figures 2D–F). To evaluate whether circ_0006677 influences NSCLC cell metabolism, we performed glucose assays and lactate assays. Overexpression of circ_0006677 significantly decreased the glucose consumption and lactic acid production in A549 and H1299 cells (Figures 2G,H). Taken together, our data suggested that circ_0006677 plays tumor-suppressive roles in NSCLC cells.

### Circ_0006677 Is a Target of MicroRNA-578 in NSCLC Cells

**FIGURE 3:**
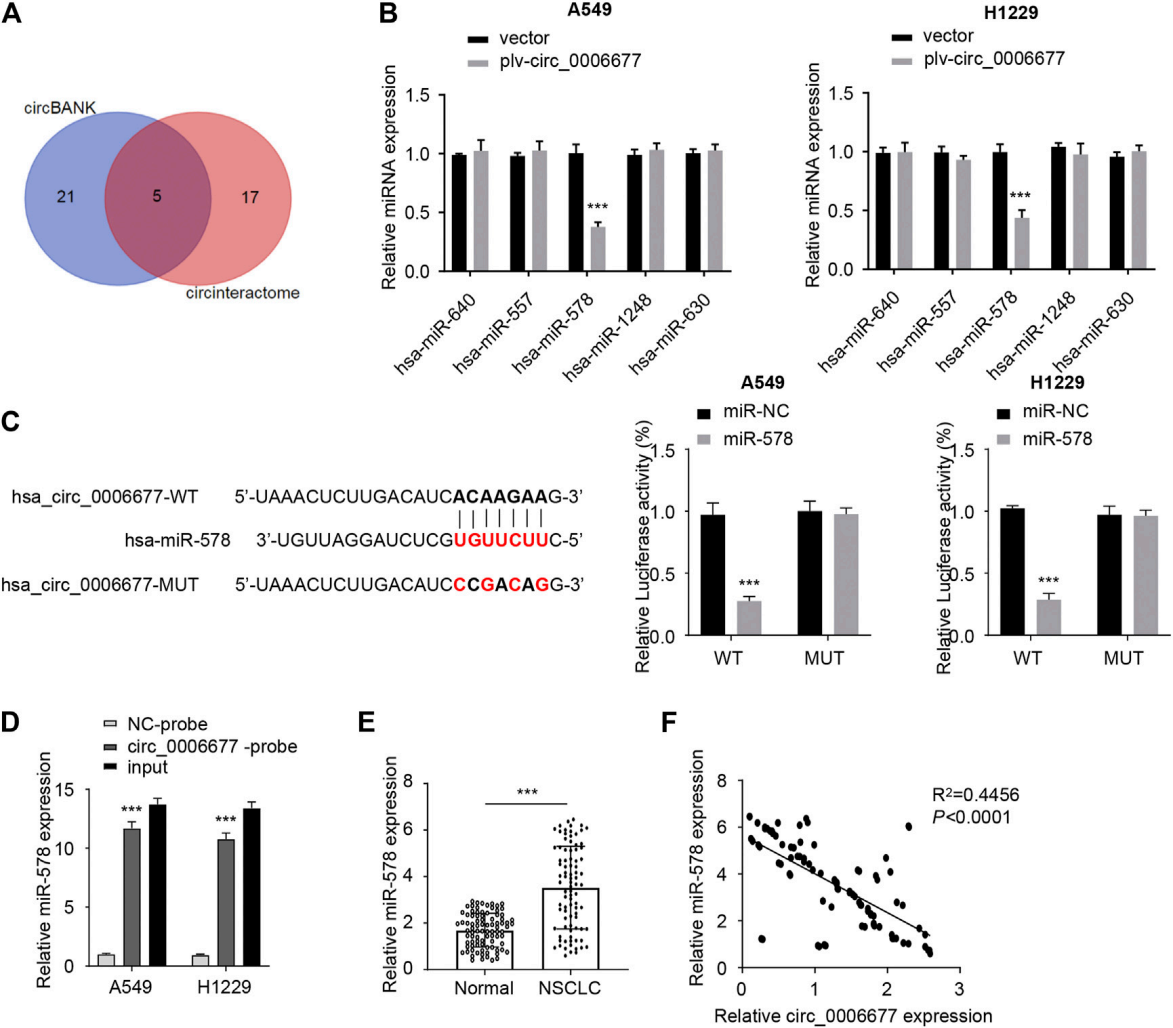
Circ_0006677 is a direct target of miR-578. **(A)** Venn diagram illustrates five overlapping miRNAs as predicted by circBank and CircInteractome databases. **(B)** qRT-PCR assays were used to detect the expression of miRNAs in A549 and H1299 cells after overexpression of circ_0006677. **(C)** Reporter assay was done to evaluate the binding between miR-578 and circ_0006677. **(D)** RNA pull-down assays were conducted to verify the binding between miR-578 and circ_000667 in A549 and H1299 cells. **(E)** The expression of miR-578 in NSCLC and normal tissues were compared using qRT-PCR assays. **(F)** Spearman correlation analysis of circ_0006677 and miR-578 in NSCLC tissues. ***p < 0.0001.

To investigate the downstream regulatory mechanism of circ_0006677 in NSCLC cells, we predicted the potential miRNAs using online prediction tools. As a result, we identified 5 miRNAs (including hsa-miR-640, hsa-miR-578, hsa-miR-557, hsa-miR-1248, and hsa-miR-630) that might bind to circ_0006677 (Figure 3A). Subsequent qRT-PCR assays showed that the expression of miR-578 (but not the remaining miRNAs) was significantly downregulated after overexpression of circ_0006677 in A549 and H1299 cells (Figure 3B). Luciferase reporter assay also confirmed that miR-578 overexpression could decrease the luciferase activity of wild-type circ_0006677 in A549 and H1299 cells. However, the inhibitory effect disappeared after mutation of the predicted miR-578 binding site was generated (Figure 3C). RNA pull-down assays further confirmed that miR-578 could bind directly to the endogenous circ_0006677 (Figure 3D). To examine the role of miR-578 in NSCLC, we first detected the expression of miR-578 in 88 pairs of NSCLC tissues and normal tissues using qRT-PCR assays. Our results demonstrated that the levels of miR-578 were significantly upregulated in NSCLC tissues compared with adjacent normal tissues (Figure 3E). Correlation analysis suggested that the expression of circ_0006677 was negatively correlated with miR-578 expression (Figure 3F). Taken together, our results suggested that circ_0006677 is a direct target of miR-578 in NSCLC cells.

### Knockdown of MicroRNA-578 Inhibits the Proliferation, Migration, Invasion, and Glycolysis of Non-Small-Cell Lung Cancer Cells

**FIGURE 4:**
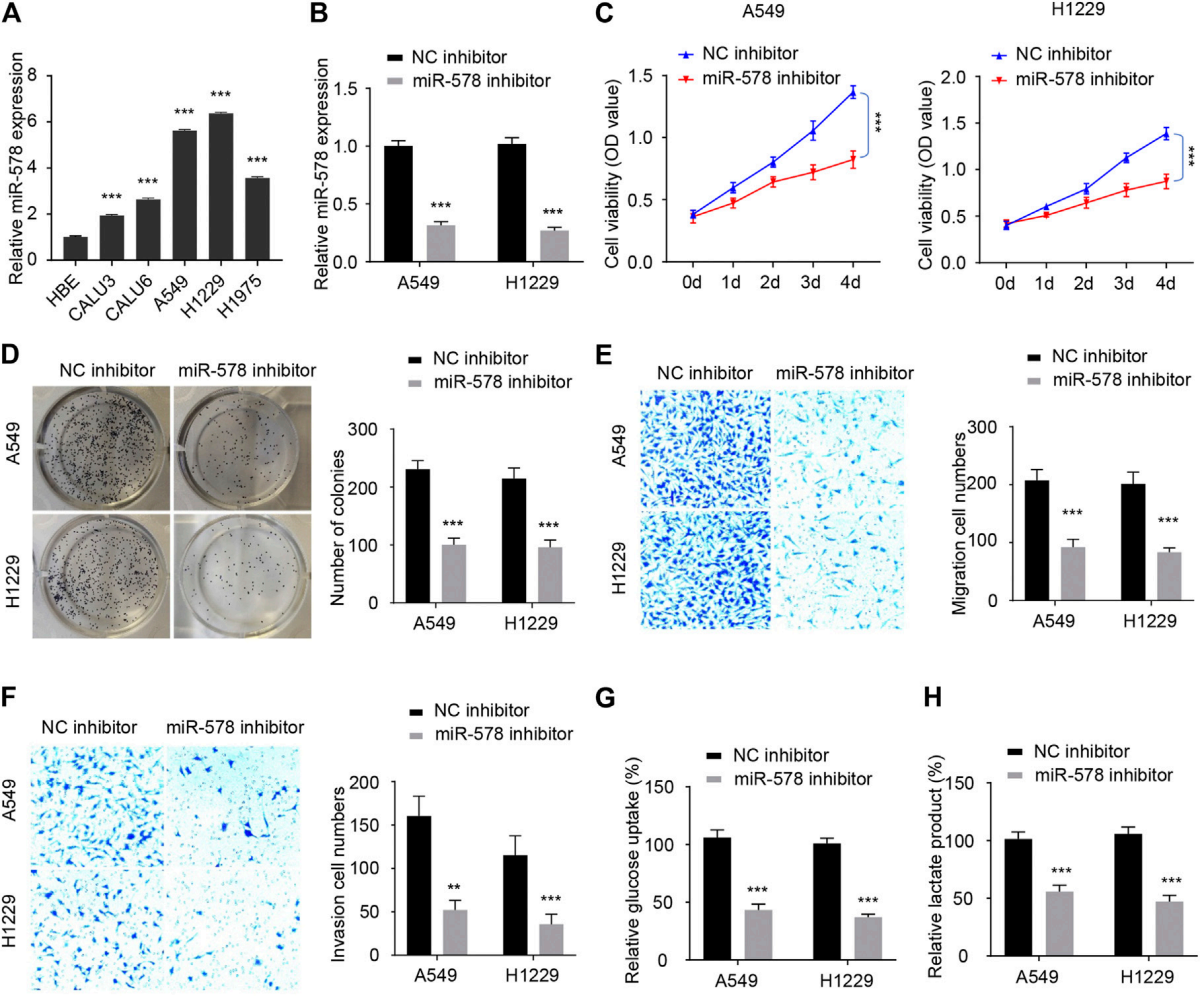
Knockdown of miR-578 expression inhibits NSCLC cell proliferation, migration, invasion, and glycolysis. **(A)** Comparison of miR-578 expression in NSCLC cell lines and HBE with qRT-PCR analysis. **(B)** qRT-PCR assays were used to examine miR-578 expression in A549 and H1299 cells transfected with miR-578 inhibitor or the respective control. **(C–H)** Cell proliferation assays **(C)**, colony formation assays **(D)**, cell migration assays **(E)**, invasion assays **(F)**, glucose consumption assays **(G)**, and lactic acid assays **(H)** of A549 and H1299 cells transfected as indicated. ***p < 0.0001.

To explore whether miR-578 exhibits a tumor-promoting role in NSCLC cells, we analyzed the expression of miR-578 in NSCLC cell lines and HBE. We validated an increased expression of miR-578 in NSCLC cells compared to HBE (Figure 4A). Relatively higher levels of miR-578 were found in A549 and H1299 cells, where circ_0006677 was expressed at low levels (Figure 1D).

Our qRT-PCR experiments showed that the transfection with miR-578 inhibitor significantly decreased the level of miR-578 in A549 and H1299 cells (Figure 4B). Cellular functional assays demonstrated that downregulation of miR-578 significantly inhibited the proliferation of A549 and H1299 cells (Figure 4C). Similarly, the colony formation, migration, and invasive abilities were also remarkably reduced by miR-578 silencing in A549 and H1299 cells (Figures 4D–F). Furthermore, transfection with miR-578 inhibitor significantly attenuated glucose consumption and lactate production in A549 and H1299 cells (Figures 4G,H). These results suggested that miR-578 promotes malignant properties and glucose metabolism in NSCLC cells.

### MicroRNA-578 Negatively Regulates the Expression of SOSC2

**FIGURE 5:**
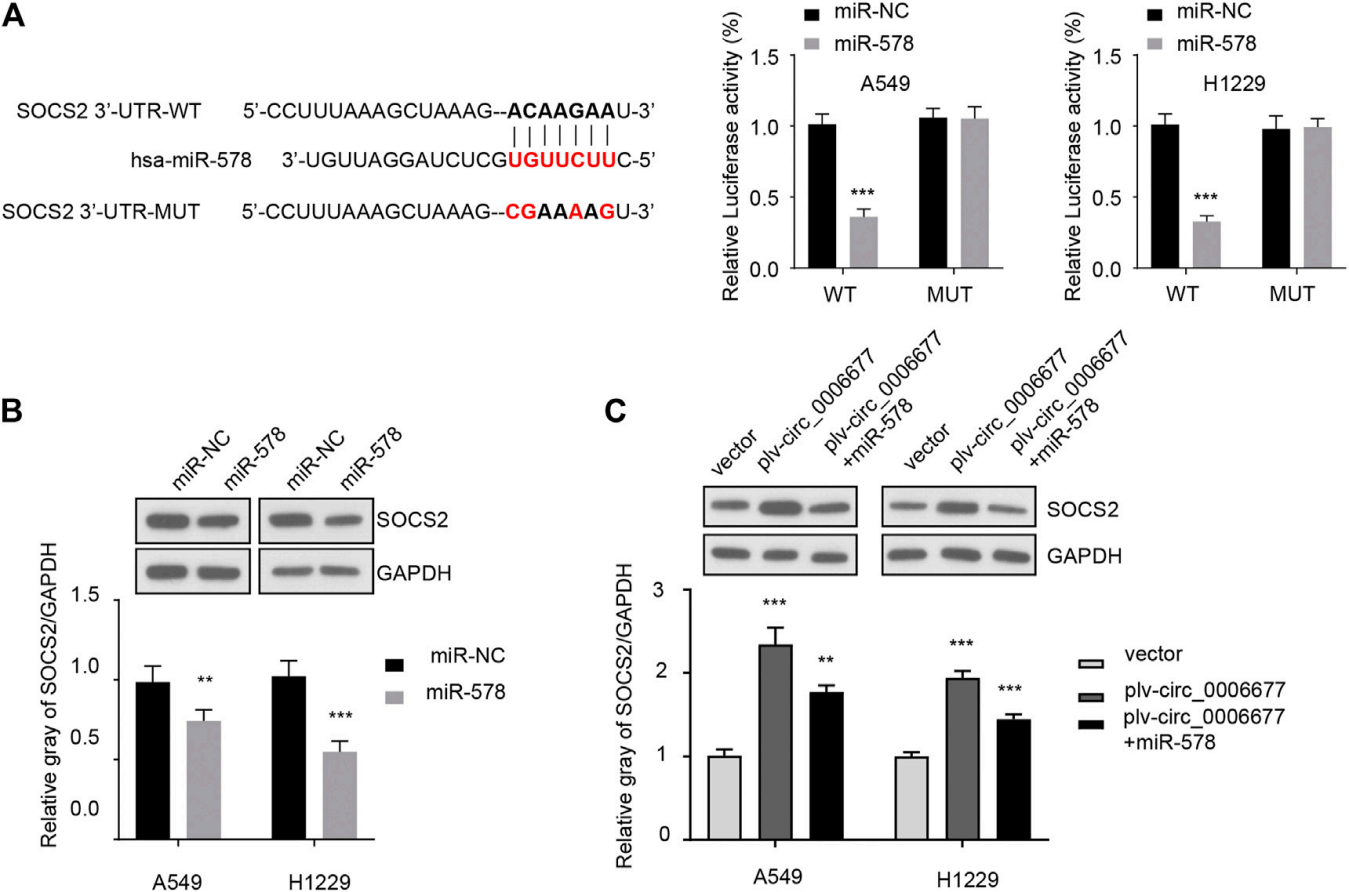
MiR-578 directly targets SOCS2 in NSCLC cells. **(A)** Reporter assays were used to evaluate the binding between miR-578 and 3′-UTR of *SOCS2* mRNA in A549 and H1299 cells. **(B)** Western blot assays were performed to examine the protein expression of SOCS2 in A549 and H1299 cells transfected with miR-578 mimic or control mimic. **(C)** Western blot assays showed that miR-578 attenuated the effects of circ_0006677 on SOCS2 protein expression in A549 and H1299 cells. ***p < 0.0001.

SOCS2, a member of the SOCS family, participates in a classical negative feedback system to inhibit cytokine signal transduction (Ricobautista et al., 2006; Das et al., 2017; Chhabra et al., 2018; Tong et al., 2019). Using the TargetScan database, we identified a binding site of miR-578 in the 3′-untranslated region (3′-UTR) of *SOCS2* mRNA. Thus, we explored whether the tumor-promoting role of miR-578 was mediated by the suppression of SOSC2. Overexpression of miR-578 significantly decreased the luciferase reporter activity of wild-type *SOCS2* 3′-UTR in A549 and H1299 cells (Figure 5A). However, these effects were abolished by mutations in the putative miR-578-binding site within the *SOCS2* 3′-UTR sequence (Figure 5A).

Western blot analysis demonstrated the inhibitory effects of miR-578 on SOCS2 protein expression in A549 and H1299 cells (Figure 5B). In addition, overexpression of circ_0006677 significantly increased the protein expression level of SOCS2 in A549 and H1299 cells, while co-transfection with miR-578 mimic reversed the effects of circ_0006677 overexpression on SOCS2 expression (Figure 5C). These findings suggested that circ_0006677 upregulates SOCS2 expression in NSCLC cells through sponging miR-578.

### Circ_0006677 Represses Non–Small-Cell Lung Cancer Progression Through Regulating the MicroRNA-578/SOCS2 Axis

**FIGURE 6:**
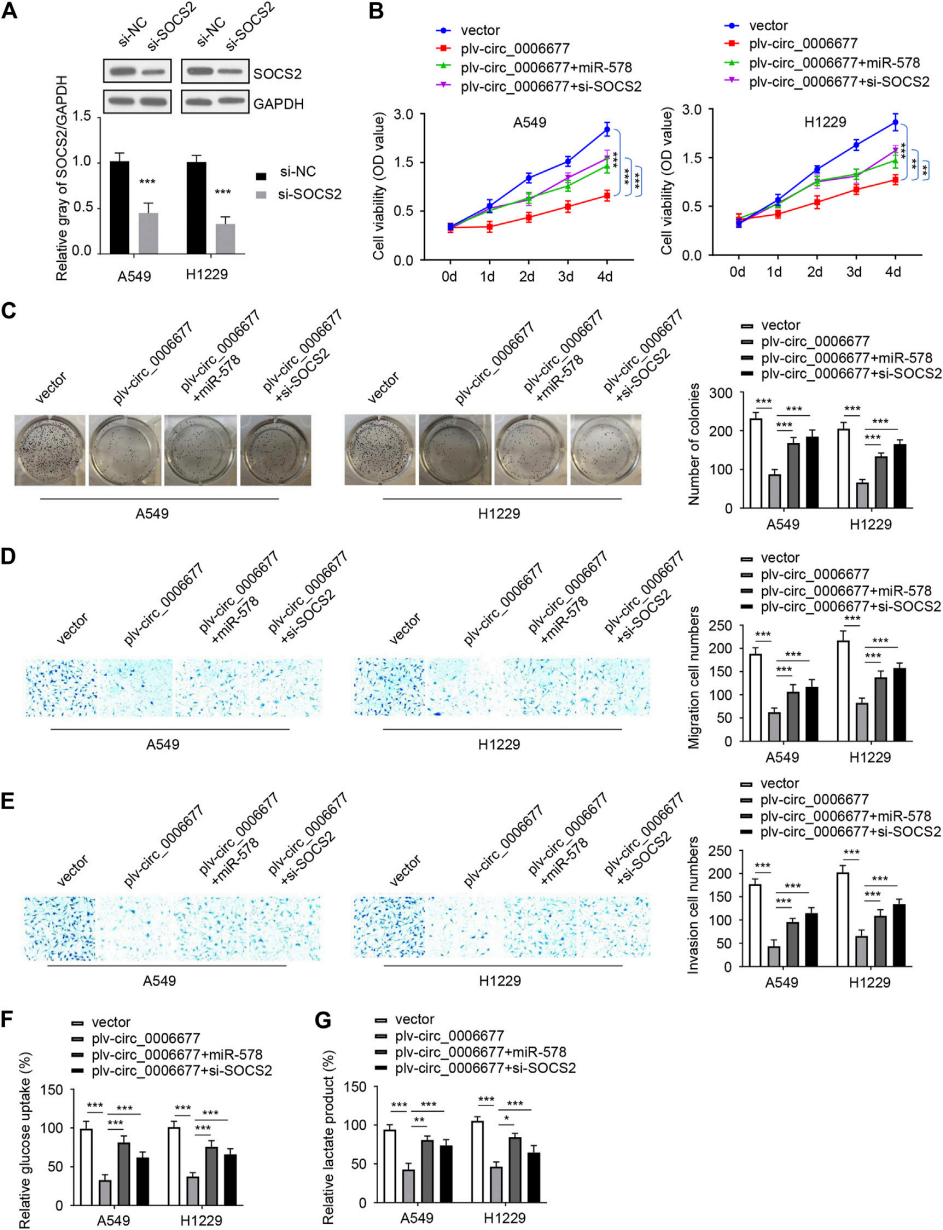
Circ_0006677 regulates NSCLC progression by the miR-578/SOCS2 axis. **(A)** Western blot assays of SOCS2 expression in A549 and H1299 cells transfected as indicated. **(B–G)** Cell proliferation assays **(B)**, colony formation assays **(C)**, cell migration assays **(D)**, invasion assays **(E)**, glucose consumption assays **(F)**, and lactic acid assays **(G)** of A549 and H1299 cells transfected as indicated. ***p < 0.0001.

To further explore whether circ_0006677 regulates NSCLC progression *via* the miR-578/SOCS2 axis, we conducted rescue experiments by transfecting NSCLC cells with the circ_0006677 expression vector, along with miR-578 mimic (or SOCS2 siRNA). As expected, overexpression of circ_0006677 significantly inhibited the proliferation, colony formation, migration, and invasion of A549 and H1299 cells; however, either miR-578 overexpression or knockdown of SOCS2, could partly restore these phenotypes of A549 and H1299 cells (Figures 6A–E). Similarly, either miR-578 overexpression or SOCS2 silencing reversed the inhibitory effects of circ_0006677 overexpression on glucose consumption and lactate production (Figures 6F,G). Collectively, our finding provided the first evidence that circ_0006677 regulates SOCS2 expression through miR-578, thereby inhibiting the progression and glucose metabolism of NSCLC.

### Circ_0006677 Suppresses Non–Small-Cell Lung Cancer Growth *in vivo*

**FIGURE 7:**
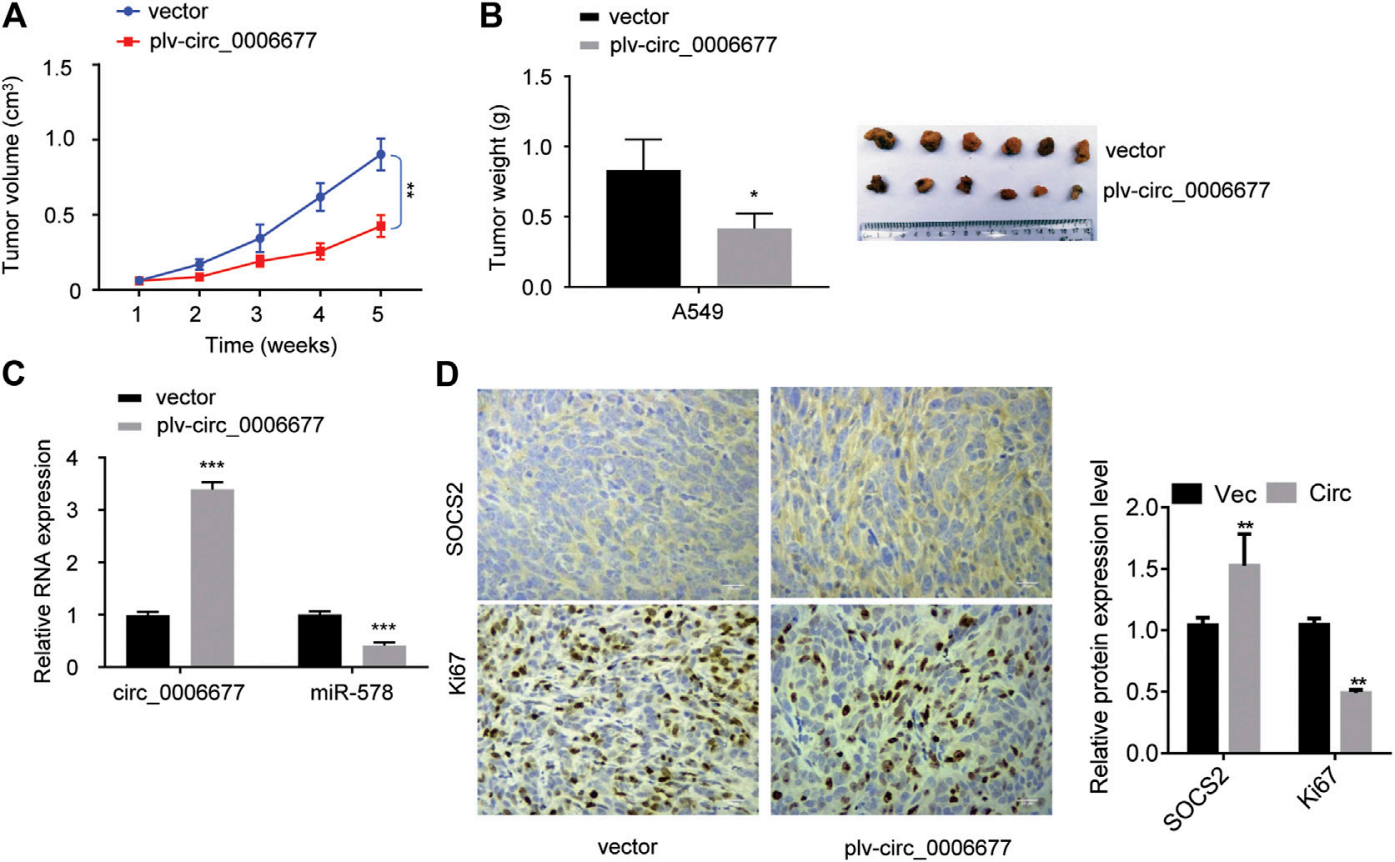
Circ_0006677 inhibits NSCLC growth *in vivo*. **(A, B)** The volume **(A)** and weight **(B)** of the xenografts derived from mice inoculated with A549 cells overexpressing c_0006677 or the control cells. **(C)** qRT-PCR analysis was used to detect circ_0006677 expression in the xenografts derived from mice inoculated with A549 cells overexpressing c_0006677 or the control cells. **(D)** Left: Immunostaining of Ki-67 and SOCS2 expression in the xenografts derived from mice inoculated with A549 cells overexpressing c_0006677 or the control cells, scale bar: 25 μm. Right: quantitative analysis of immunostaining data. *p < 0.05, **p < 0.001, ***p < 0.0001.

To investigate the role of circ_0006677 *in vivo*, we established the xenograft mouse models by subcutaneously inoculating the stable A549 cells overexpressing circ_0006677 or control cells into nude mice. The volume and weight of tumors in mice bearing A549 cells overexpressing circ_0006677 were markedly lower than those in mice bearing control A549 cells (Figures 7A,B). The qRT-PCR analysis validated that the levels of circ_0006677 were significantly higher, whereas miR-578 expression was significantly lower in circ_0006677–overexpressing tumors than in the control group (Figure 7C). Immunohistochemistry staining revealed that Ki-67 expression was decreased, while the SOCS2 level was increased in the circ_0006677–overexpressing group compared with the control group (Figure 7D), confirming the inhibitory effects of circ_0006677 on NSCLC growth *in vivo*.

## Discussion

CircRNAs have been considered as important regulators in cancer progression (Dou et al., 2016; Qin et al., 2016; Wan et al., 2016; Chen et al., 2017; Bach et al., 2019). Elucidating the molecular association between circRNA dysregulation and NSCLC progression is of great importance to identify crucial diagnostic and therapeutic targets of this cancer. In our current study, we demonstrated for the first time that circ_0006677 acts as a key tumor suppressor to inhibit NSCLC progression and metabolic reprogramming *via* mediating the miR-578/SOCS2 pathway. Thus, circ_0006677/miR-578/SOCS2 signaling could be a potential target for NSCLC diagnosis and treatment.

NSCLC is the most common type of lung cancer, accounting for 84% of all lung cancer cases (Sève et al., 2010). Compared with the traditional biomarkers (such as oncogene mutants and miRNAs), circRNAs exhibit cell- and tissue-specific patterns (Bach et al., 2019). Unlike linear RNAs, circRNAs are more stable in tissues and plasma due to the inaccessibility to degradative enzymes (Yao et al., 2017; Bach et al., 2019; Dong et al., 2020). In our study, using the GEO dataset, we showed that circ_0006677 expression was reduced in NSCLC tissues. We further confirmed the downregulation of circ_0006677 in NSCLC samples and NSCLC cell lines, and low expression of circ_0006677 was associated with unfavorable clinicopathologic features (including larger tumor size, advanced tumor stage, and the presence of metastasis) and worse prognosis in NSCLC patients, suggesting that circRNAs might be an ideal diagnostic and prognostic biomarker for NSCLC.

To date, little is known about the cellular function of circ_000667 in NSCLC cells. Here, our *in vitro* and *in vivo* experiments clearly showed that circ_0006677 plays a critical tumor-suppressive role in NSCLC progression by inhibiting the proliferation, migration, invasion, and glucose metabolism of NSCLC cells, as well as the growth properties of NSCLC cells in a subcutaneous tumor xenograft models, indicating that circ_0006677 might be a new therapeutic target for NSCLC treatment.

Recent studies have suggested that circRNAs may function as miRNA sponges to modulate cancer development (Bach et al., 2019). In our present study, we demonstrated the negative associations between circ_0006677 and miR-578 expressions in NSCLC tissues. Consistently, our results showed that miR-578 attenuated the ability of circ_0006677 to enhance the aggressive properties and glucose metabolism of NSCLC cells. In summary, our results supported that circ_0006677 inhibits NSCLC progression through sponging miR-578.

Abnormal expression of miR-578 has been linked to cancer progression (Chen et al., 2020; Ji et al., 2020; Wu et al., 2020). However, the function of miR-578 in NSCLC remains unknown. In our study, we have provided evidence to show that knockdown of miR-578 could significantly prevent the proliferation, migration, invasion, and glucose metabolism of NSCLC cells, indicating the tumor-promoting functions of miR-578 in NSCLC.

SOCS2 suppresses the cytokine-induced signaling transduction and its downregulation is observed in many cancers (Ricobautista et al., 2006; Das et al., 2017; Chhabra et al., 2018). Here, we have revealed that SOCS2 represses the progression of NSCLC, and it serves as a direct target of miR-578. The protein expression of SOCS2 was elevated by circ_0006677 overexpression and inhibited by miR-578 in NSCLC cells. Hence, circ_0006677 can sponge miR-578 to induce SOCS2 expression, and this ceRNA network might be a new therapeutic target for the treatment of NSCLC. Moreover, our results suggested that circ_0006677 inhibited the growth of NSCLC by inhibiting glucose consumption and lactate production through regulating SOCS2 expression. These data further confirm that SOCS2 is essential for circ_0006677–mediated tumor suppression in NSCLC.

In conclusion, circ_0006677 is significantly downregulated and negatively associated with poor outcomes in NSCLC patients. Furthermore, circ_0006677 regulates the Warburg effect and NSCLC progression *via* increasing SOCS2 expression through sponging miR-578. Our findings uncover a mechanistic role for the circ_0006677/miR-578/SOCS2 signaling pathway in NSCLC progression and metabolic reprogramming, providing a potential therapeutic strategy for patients with NSCLC.

## Data Availability

The original contributions presented in the study are included in the article/Supplementary Material; further inquiries can be directed to the corresponding authors.
