# Supplementary material for: CircRNA circ_0006677 Inhibits the Progression and Glycolysis in Non–Small-Cell Lung Cancer by Sponging miR-578 and Regulating SOCS2 Expression
Source: Front Pharmacol. 2021 May 13;12:657053. doi: 10.3389/fphar.2021.657053 (PMC8155686; doi:10.3389/fphar.2021.657053)
Supplement: Supplementary file 1 [file Table1.DOCX]

| Factor |  | circ_0006677 expression | | *P* value |
| --- | --- | --- | --- | --- |
|  |  | Low (n=44) | High (n=44) |  |
| Age |  |  |  | 0.286 |
|  | ≤60 | 24 | 19 |  |
|  | ＞60 | 20 | 25 |  |
| Sex |  |  |  | 0.514 |
|  | Male | 28 | 25 |  |
|  | Female | 16 | 19 |  |
| Tumor differentiation | |  |  | 0.197 |
|  | I | 16 | 22 |  |
|  | II | 28 | 22 |  |
| Tumor size |  |  |  | 0.001 |
|  | ≤2cm | 10 | 26 |  |
|  | ＞2cm | 32 | 18 |  |
| T classification | |  |  | 0.029 |
|  | T1-T2 | 15 | 25 |  |
|  | T3-T4 | 27 | 17 |  |
| N classification | |  |  | 0.016 |
|  | N0-N1 | 17 | 28 |  |
|  | N2-N3 | 25 | 14 |  |
| Clinical stage | |  |  | 0.016 |
|  | I/II | 16 | 27 |  |
|  | III/IV | 26 | 15 |  |
| Lymph node metastasis | |  |  | 0.012 |
|  | Yes | 22 | 32 |  |
|  | No | 22 | 10 |  |
